# Alteration of the Thymic T Cell Repertoire by Rotavirus Infection Is Associated with Delayed Type 1 Diabetes Development in Non-Obese Diabetic Mice

**DOI:** 10.1371/journal.pone.0059182

**Published:** 2013-03-15

**Authors:** Nicole L. Webster, Christel Zufferey, Jessica A. Pane, Barbara S. Coulson

**Affiliations:** Department of Microbiology and Immunology, The University of Melbourne, Parkville, Victoria, Australia; University of California, San Francisco, United States of America

## Abstract

Rotaviruses are implicated as a viral trigger for the acceleration of type 1 diabetes in children. Infection of adult non-obese diabetic (NOD) mice with rotavirus strain RRV accelerates diabetes development, whereas RRV infection in infant NOD mice delays diabetes onset. In this study of infant mice, RRV titers and lymphocyte populations in the intestine, mesenteric lymph nodes (MLN) and thymus of NOD mice were compared with those in diabetes-resistant BALB/c and C57BL/6 mice. Enhanced intestinal RRV infection occurred in NOD mice compared with the other mouse strains. This was associated with increases in the frequency of CD8αβ TCRαβ intraepithelial lymphocytes, and their PD-L1 expression. Virus spread to the MLN and T cell numbers there also were greatest in NOD mice. Thymic RRV infection is shown here in all mouse strains, often in combination with alterations in T cell ontogeny. Infection lowered thymocyte numbers in infant NOD and C57BL/6 mice, whereas thymocyte production was unaltered overall in infant BALB/c mice. In the NOD mouse thymus, effector CD4^+^ T cell numbers were reduced by infection, whereas regulatory T cell numbers were maintained. It is proposed that maintenance of thymic regulatory T cell numbers may contribute to the increased suppression of inflammatory T cells in response to a strong stimulus observed in pancreatic lymph nodes of adult mice infected as infants. These findings show that rotavirus replication is enhanced in diabetes-prone mice, and provide evidence that thymic T cell alterations may contribute to the delayed diabetes onset following RRV infection.

## Introduction

Rotaviruses are the major etiologic agents of severe acute infantile gastroenteritis [1]. Environmental factors including viruses are implicated in the rising incidence of type 1 diabetes, an autoimmune disease resulting in T cell-mediated destruction of insulin-producing cells within the pancreas. Diabetes onset is preceded by development of pancreatic islet autoimmunity, including autoantibodies that mark progression towards diabetes [2], [3]. Correlations between rotavirus infection and exacerbations in the level of islet autoantibodies in children genetically at-risk of developing diabetes have been observed, suggesting that rotaviruses may play a role in diabetes development [4], [5].

Non-obese diabetic NOD/Lt (NOD) mice spontaneously develop diabetes as they age and are a commonly used model for human diabetes [6], [7]. Infection of older adult NOD mice with pre-existing islet autoimmunity by monkey rotavirus strain RRV accelerates diabetes onset, whereas RRV infection of infant NOD mice delays diabetes onset [8], [9]. RRV is present in the intestine, liver, pancreas, spleen and blood of infant NOD mice, but does not reach the pancreas in the adults. While these findings show the potential for rotaviruses to either accelerate or delay diabetes, the precise nature of the virus and host factors involved is unclear. Identifying how diabetes can be delayed is necessary to devise strategies for delaying the age of diabetes onset in children and substantially improving their quality of life.

Intestinal T lymphocytes play an important role in the rotavirus-specific immune response. Intraepithelial lymphocytes (IEL) comprise 3–10% of all cells residing within the intestinal epithelium [10]. CD8αβ TCRαβ IEL recognize non-self antigen presented by conventional MHC class I molecules [11], secrete Th1 cytokines (eg. IFNγ) and are cytotoxic during acute viral infection [12], [13], [14]. Rotavirus-specific CD8^+^ T cells present in the IEL compartment and the mesenteric lymph nodes (MLN) at 6 days after infection of adult C57BL/6 mice show direct anti-viral activity for timely resolution of primary infection [15]. CD4^+^ T cells are essential for development of the rotavirus-specific IgA response in the intestine [15], and are the only cell type sufficient to confer protection from re-infection [16]. The programmed cell death-ligand 1 (PD-L1) is a costimulatory molecule expressed on a range of cell types including T cells and epithelial cells following stimulation with IFNγ [17]. PD-L1 expression is important for T cell activation, cytokine production and virus-specific T cell responses [18], [19]. During coxsackievirus B3 or lymphocytic choriomeningitis virus infection, PD-L1 expressed by lymphocytes inhibits diabetogenic CD8^+^ T cell expansion in NOD mice, delaying diabetes development [20]. It is possible that PD-L1 also may play a role in the delayed diabetes onset in NOD mice following rotavirus infection. However, the dynamics of PD-L1 expression on CD8^+^ IEL during the acute phase of rotavirus infection has not been investigated.

Type 1 diabetes reflects a loss of tolerance to self-antigen. In central tolerance, potentially autoreactive lymphocytes emerging in the thymus are eliminated. This process is incomplete so additional peripheral tolerance mechanisms exist, including immune response suppression by regulatory T cells (Treg) via TGFβ and IL-10 production. Virus infection of the thymus has the potential to affect tolerance and diabetes development [21], [22], [23]. Like RRV, reovirus infection delays diabetes onset in infant NOD mice. Proposed mechanisms for the reovirus-mediated delay include infection of the thymus resulting in loss of autoreactive T cells and passive tolerance [24], [25]. Although untested to date, it is plausible that rotavirus might delay murine diabetes through thymic infection and effects on the T cell repertoire, particularly as infectious RRV is present in the thymus of infant rats [26].

The main aim of the current study was to investigate the effect of RRV infection on T lymphocytes in the intestine, lymph nodes and thymus of infant NOD, C57BL/6 and BALB/c mice. It was demonstrated that intestinal RRV infection is enhanced in NOD mice over the other strains, in association with increases in CD8αβ TCRαβ IEL frequency and T cell numbers in MLN. Thymic RRV infection, shown here for the first time in mice, was associated with altered T cell ontogeny. NOD mice alone showed an increased ratio of CD4^+^ Treg to CD4^+^ T effector cells (Teff) produced in the thymus following RRV infection. This may prolong suppression of inflammatory T cell responses in the pancreatic lymph node (PLN) and contribute to the delayed diabetes onset.

## Materials and Methods

### Mice

Mice obtained from the Animal Resource Centre, Western Australia were bred and housed in the Biological Research Facility of the Department of Microbiology and Immunology at the University of Melbourne in individually ventilated micro-isolator cages under specific pathogen free conditions as previously described [8].

### Ethics statement

Principles of laboratory animal care' (NIH publication no. 85–23) and the ‘Australian Code of Practice for the Care and Use of Animals for Scientific Purposes (2004)’ were followed. All procedures were conducted in accordance with protocols approved by the Animal Ethics Committee of The University of Melbourne (ID 1011742).

### Mouse inoculation with RRV and diabetes monitoring

Monkey rotavirus RRV was cultivated in MA104 cells (American Type Culture Collection), purified by glycerol gradient ultracentrifugation, and titrated for infectivity as before [27], [28]. Five day-old mice were inoculated by oral feeding with 10 µl of either 50 mM Tris HCl pH 7.4 containing 150 mM NaCl and 5 mM CaCl_2_ (TSC; mock-inoculated controls) or TSC containing 6×10^6^ fluorescent forming cell units (FCFU) of RRV, as described previously [8]. In indicated experiments, female NOD mice (aged-matched to females given RRV as infants where indicated), were infected at 12 weeks of age by oral gavage with 2×10^6^ FCFU of RRV in 200 µl of TSC, as before [8], [9]. Mice reaching 6 weeks of age were monitored weekly for diabetes onset by blood or urinary glucose testing, as described previously [8], [9].

### Assay for antibodies to rotavirus

Titers of rotavirus antibodies in mouse sera were determined by EIA with RRV antigen on the solid phase, as previously described [8]. Seroconversion was defined as a four-fold increase in titer. All dams were negative for serum antibodies to RRV prior to experimentation. All infant mice infected with RRV that reached 14 days of age were tested at this time and shown to seroconvert to RRV.

### Detection of infectious RRV and determination of RRV titer

The procedures for collection and processing of tissues have been described previously [8] apart from the thymus and MLN, which were prepared as a 10% (wt/vol) homogenate in TSC. Infectious RRV was detected by culture amplification in MA104 cells for 72 h followed by antigen capture EIA, as before [29], [30]. Virus titers were determined by MA104 cell culture and indirect immunofluorescent staining, as described previously [28], [29].

### Cell isolation from thymus, MLN, PLN and spleen

Tissues were mechanically disrupted between glass slides. Released cells were filtered through 70 uM nylon mesh and washed with either RPMI containing 10% (vol/vol) fetal bovine serum (FBS; RF10) for cell culture or with FACS buffer (1% (vol/vol) FBS in Ca^2+^-and Mg^2+^-free PBS with 0.01% (wt/vol) NaN_3_) for antibody staining and flow cytometric analysis. Cell counts were determined with a hemocytometer.

### IEL isolation

This method was adapted from [31]. Briefly, 0.5 cm to 1 cm long pieces of small intestine were stirred in Ca^2+^- and Mg^2+^-free HBSS containing 10 mM HEPES, 2% (vol/vol) FBS, 0.5 mM EDTA and 2 mM dithiothreitol at 37°C for 30 min, followed by two further incubations in the absence of dithiothreitol. Detached cells were counted prior to flow cytometric analysis.

### Enzyme-linked immunospot assay (ELISPOT)

MLN cells (2×10^5^/well) cultured in RF10 containing 10% (vol/vol) FBS and IL-2 at 40 U/ml were stimulated at 20 µg/ml with a peptide corresponding to amino acids 5 to 13 of RRV VP7 (GenScript) [32], or 50 ng/ml PMA and 500 ng/ml ionomycin as a positive control, for 48 h. Cells responding to the peptide were identified with mouse IFNγ ELISPOT and 2-amino-9-ethylcarbazole (AEC) kits (BD). Stained cells were viewed and counted by microscopy.

### Flow cytometric analysis of isolated cells

Cells (1×10^6^) were resuspended in FACS buffer for antibody staining. IEL were stained with conjugated antibodies as follows: anti-CD3 (500A2)-Alexa700, anti-CD4 (RM4-5)-Pacific Blue, anti-CD8α (53-6.7)-PerCP, anti PD-L1 (MIH5)-phycoerythrin (PE) (all from BD), anti-T cell receptor (TCR) β-chain (H57-597)-PE-Cy7 (Biolegend, San Diego, CA), anti-TCRγδ (GL-3)-allophycocyanin (APC) and anti-CD8β (ebioH35-17.2)-biotin (eBioscience). Streptavidin APC-Alexa 750 (Invitrogen) was used as secondary antibody. Thymus, spleen and lymph node cells were stained with anti-CD3, anti-CD8α, anti-CD4, anti-CD25 (PC61)-APC and anti-Foxp3 (FJK-16 s)-PE. The Foxp3 staining buffer set (eBioscience) was used for intracellular staining. IFNγ intracellular staining was performed using anti-IFNγ (XMG1.2)-FITC and Cytofix/Cytoperm Plus kit (BD). Acquisition of at least 5×10^5^ cells was performed on the LSRII (BD) and data analysed with FlowJo 8.7.1 (Tree Star). The absolute number of each cell subset was calculated using the frequency of the subset of interest obtained by flow cytometry and the total number of cells determined by hemocytometer counts.

### Immunohistochemistry and histology

Thymuses were fixed in 2% (vol/vol) paraformaldehyde and embedded in paraffin. Sections (4 µm) were cut, deparaffinised, blocked with 10% (vol/vol) goat serum in PBS for 30 min and reacted for 30 min with either rabbit anti-RRV antiserum or normal rabbit serum (rotavirus antibody-negative), each diluted 1 in 500 in PBS containing 0.5% (vol/vol) FBS. Endogenous peroxidase activity was blocked with 1 part 3% (vol/vol) H_2_O_2_ solution in 4 parts methanol for 20 min. Bound antibody was detected with HRP-conjugated goat anti-rabbit antibody (Millipore) and SIGMA*FAST*™. The location of RRV-positive cells in serial sections counterstained with hematoxylin was identified by microscopy, and images collected using Leica LAS software v 3.8.

### PLN cell proliferation

Isolated cells were serially diluted from 4×10^5^ cells/well in U-bottomed 96 well plates (Nunc) and cultured with anti-CD3 (2 µg/ml; BD), anti-CD28 (1 µg/ml; BD) and IL-2 (20 U/ml; Peprotech) in RF10. Controls comprised cells alone and cells with IL-2 alone. Cultures were incubated for 72 h at 37°C with 5% (vol/vol) CO_2_. Cell proliferation was assessed by addition of ^3^H-thymidine at 1 µCi/well for the final 24 h of incubation. Cells were collected onto glass fibre filters using an automated cell harvester (Skatron Instruments) and β-emission recorded by a liquid scintillation counter (Packard Bell) in counts/min.

#### Statistical analysis

Student's t-test, with Welch's correction as appropriate, was used to assess statistical significance, which was set at the 95% level. On graphs, error bars indicate the SD unless otherwise indicated.

## Results

### NOD mice showed the greatest extent of intestinal RRV replication

**Figure 1:**
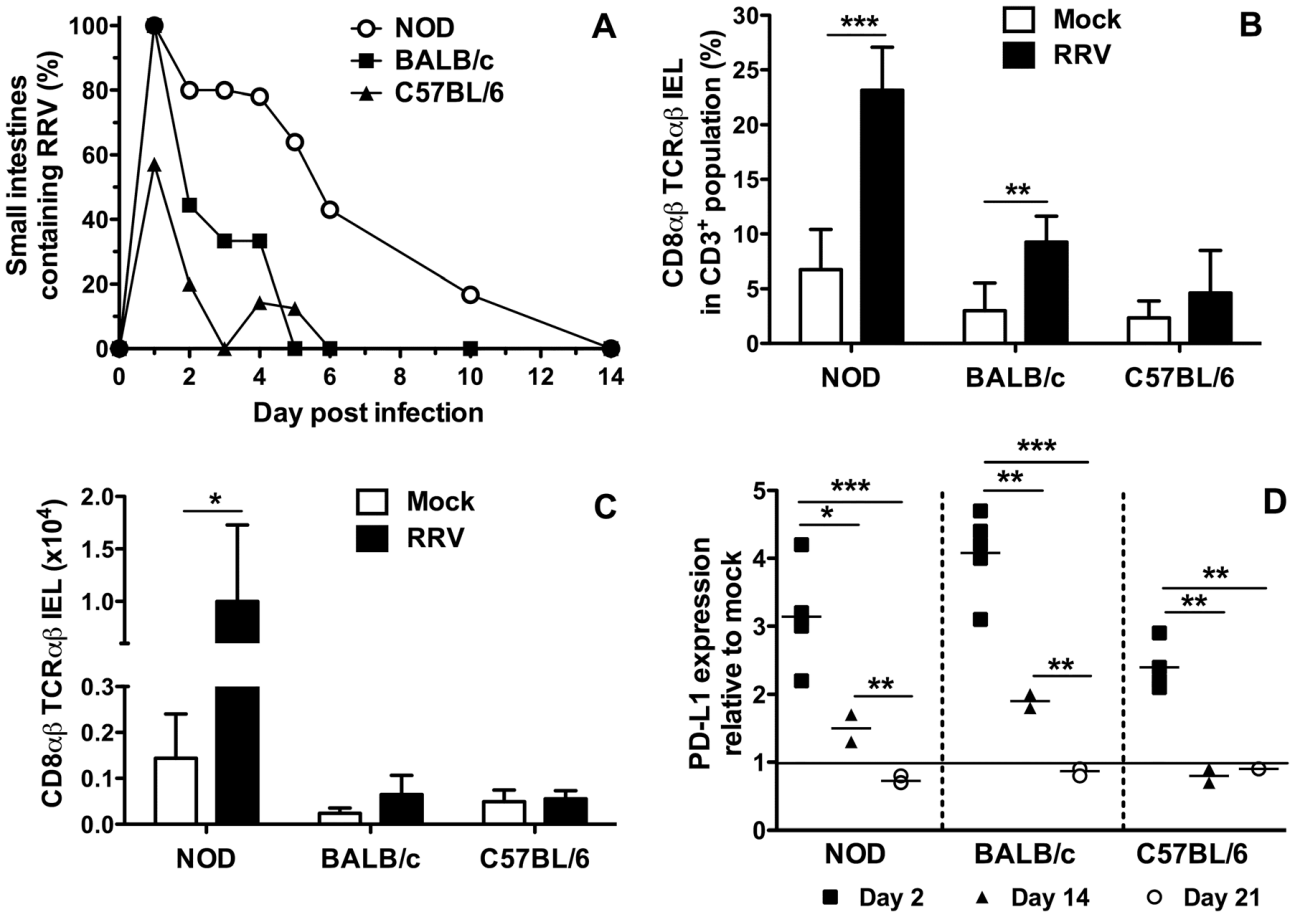
RRV replication and IEL responses in the small intestine of RRV-infected infant mice. A, Proportion of intestines containing infectious RRV following inoculation of 5 day-old NOD, BALB/c and C57BL/6 mice. Infectious RRV was detected by 72 h co-culture with MA104 cells followed by EIA. At each day post infection, intestines from 5 to 6 NOD mice were analysed. On days 1, 2, 3, 4, 5, 6 10 and 14 post infection, intestines from 8, 9, 6, 9, 6, 8, 5 and 6 BALB/c mice and 7, 5, 7, 7, 8, 9, 7, and 6 C57BL/6 mice were assayed, respectively. B, Frequency and C, absolute numbers of CD8αβ TCRαβ IEL on day 2 post mock- or RRV infection. To obtain sufficient cells, 4 to 6 separate cell pools were analysed for each inoculum. Each pool was produced from 2 to 5 (NOD), 2 to 3 (BALB/c) or 3 to 5 (C57BL/6) mice. Cell numbers from pools were normalized to represent cells obtained from a single mouse. D, PD-L1 expression by CD8αβ TCRαβ IEL in RRV-infected mice on days 2, 14 and 21 after infection. Data are expressed as the ratio of PD-L1 expression on cells from RRV-infected mice to the mean PD-L1 expression of mock-infected mice. The numbers of mice analysed and usage of cell pools for day 2 studies were as described for B and C. For days 14 and 21 post infection, each data point represents a pool of cells from 2 (NOD), 3 to 4 (BALB/c) or 2 to 3 (C57BL/6) mice. * p<0.05; ** p<0.01; *** p<0.001. Bar = mean.

RRV-infected infant NOD mice exhibit diarrhoea for 2 to 5 days, and intestinal RRV presence for up to 9 days [8]. We found here that diarrhoea following oral RRV infection of infant BALB/c and C57BL/6 mice lasted for 5 to 8 days and 1 day, respectively. At day 1 post infection all intestines from NOD (n = 6) and BALB/c mice (n = 8) contained infectious RRV, whereas only 4/7 C57BL/6 were RRV-positive (Fig. 1A). By day 3 post infection, 4/5 NOD, 2/6 BALB/c and 0/7 C57BL/6 mouse intestines remained infected. NOD mice showed the longest period of RRV presence intestinally. The reciprocal geometric mean titers of infectious RRV intestinally in NOD and BALB/c mice did not differ significantly (p>0.05), but both were significantly increased over those in C57BL/6 mice at day 1 post-infection (Table 1; p≤0.0044). NOD mice showed the greatest proportion of mice with detectable infectious RRV at all days post infection except day 1, when BALB/c mice showed an equal rate (Fig. 1A). Although intestinal RRV was detected over a similar period (4 to 5 days) in BALB/c and C57BL/6 mice, a lesser proportion of C57BL/6 than BALB/c mice showed intestinal RRV replication (Fig. 1A). The proportion of mice showing infectious RRV intestinally and their RRV titers were associated with the extent of diarrhoea observed, as BALB/c and NOD mice showed more protracted diarrhoea, greater infection rates and higher titers than C57BL/6 mice.

**Table 1:** RRV titers in homogenates of small intestines and MLN from RRV-infected infant BALB/c, NOD and C57BL/6 mice.

Day post infection	Proportion of organs in the given mouse strain from which a virus titer was obtained, and the virus titer											
	Small intestine						MLN					
	NOD		BALB/c		C57BL/6		NOD		BALB/c		C57BL/6	
	% of mice^1^	Virus titer^2^	% of mice	Virus titer	% of mice	Virus titer	% of mice	Virus titer	% of mice	Virus titer	% of mice	Virus titer
1	100 (6/6)	3,300 (510–20,000)	100 (8/8)	1,100 (240–2,600)	29 (2/7)	410 (120–1,400)	0 (0/6)	NA^3^	0 (0/8)	NA	0 (0/7)	NA
2	80 (4/5)	450 (240–960)	33 (3/9)	550 (240–960)	20 (1/5)	120	80 (4/5)	65 (23–390)	0 (0/9)	NA	0 (0/5)	NA
3	60 (3/5)	750 (480–1,200)	17 (1/6)	480	0 (0/7)	NA	40 (2/5)	85 (45–160)	33 (2/6)	45 (45–45)	0 (0/7)	NA
4	ND^4^	ND	22 (2/9)	960 (480–1,900)	14 (1/7)	480	100 (5/5)	1,300 (230–4,700)	22 (2/9)	220 (160–300)	43 (3/7)	96 (24–900)
5	ND	ND	0 (0/6)	NA	0 (0/8)	NA	80 (4/5)	1,600 (450–11,000)	33 (2/6)	500 (160–1,600)	25 (2/8)	7 (4–12)
6	ND	ND	0 (0/8)	NA	0 (0/9)	NA	0 (0/5)	NA	63 (5/8)	58 (12–180)	0 (0/9)	NA

**Figure 2:**
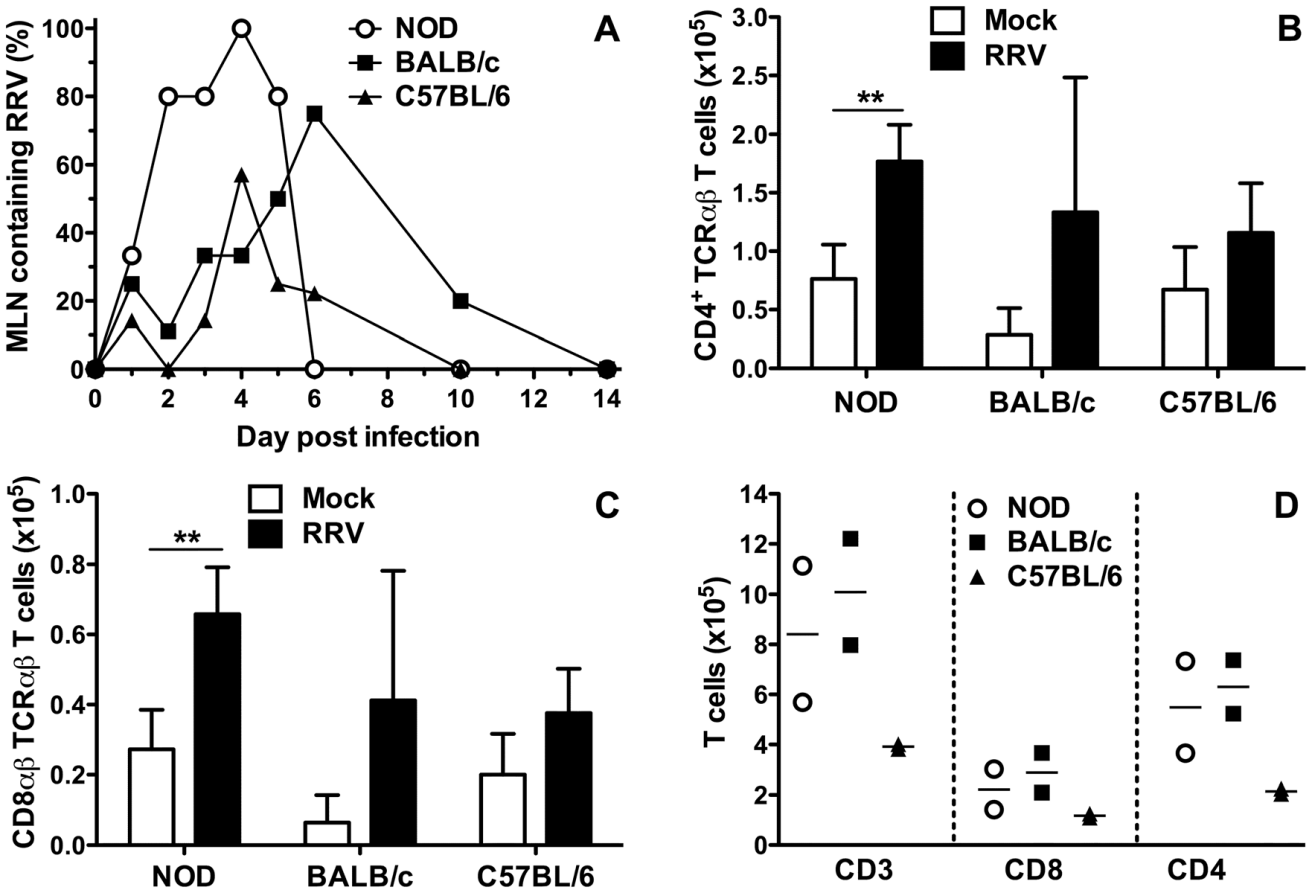
RRV replication and T cell subsets in the MLN of RRV-infected infant mice. A, Proportion of MLN containing infectious RRV. The numbers of mice analysed on each day are given in the Legend to Fig. 1A. Absolute numbers of CD4^+^ TCRαβ T cells (B) and CD8αβ TCRαβ T cells (C) at day 2 post infection are shown. To obtain sufficient cells, 4 to 5 separate cell pools were analysed for each inoculum. Each pool was produced from 4 to 5 (NOD), 3 to 4 (BALB/c) or 3 to 5 (C57BL/6) mice. Cell numbers from pools were normalized to represent cells obtained from a single mouse. D, Absolute numbers of CD3^+^, CD8^+^ and CD4^+^ T cells from RRV-infected mice at day 6 post infection. Each data point represents a pool of cells from 4 (NOD), 3 (BALB/c) or 3 to 4 (C57BL/6) mice. * p<0.05; ** p<0.01. Bar = mean.

1 Percentage (no. giving a virus titer/no. collected) of mouse organs where an infectious virus titer could be determined. All organs where a titer was measurable also showed infectious virus by co-culture (Fig. 1A, 2A).

2 Reciprocal geometric mean titer (range) of RRV in FCFU/organ. No range is given when only a single sample yielded a virus titer.

3 Not applicable. No infectious RRV was detected by titration.

4 Not done. In previous studies our group detected virus in 67% of intestines from RRV-infected NOD mice on days 4 and 5, and 40% on day 6 [8].

### Intestinal IEL responses to RRV infection in NOD, BALB/c and C57BL/6 mice

The effect of RRV infection on intestinal CD8^+^ T cells in infant mice was determined by isolation and characterisation of intestinal IEL at 2, 5, 11, 14, 21 and 35 days after RRV or mock inoculation. The frequency of CD8αβ TCRαβ IEL at day 2 was significantly increased over controls in RRV-infected NOD and BALB/c mice, but not C57BL/6 mice (p = 0.023, p = 0.0001 and p>0.05, respectively; Fig 1B). CD8αβ TCRαβ IEL numbers were significantly increased in RRV-infected NOD mice (p = 0.029), but not BALB/c or C57BL/6 mice (p>0.05; Fig 1C). RRV-infected NOD mice showed fewer CD8αβ TCRαβ IEL (mean ± SD = 1.0 ± 0.01×10^4^ cells) than mock-infected mice (mean ± SD = 2.0±0.20×10^4^ cells; p = 0.02) at day 14 post-infection (data not shown). Aside from this, no change in CD8αβ TCRαβ IEL frequency or numbers was observed in any mouse strain later than 2 days post-infection (data not shown).

PD-L1 expression on CD8αβ TCRαβ IEL of RRV-infected infant mice increased by a mean of 2.4- to 4.0-fold relative to control mice for all mouse strains at day 2 post infection (Fig. 1D; 0.0001≤p≤0.0030). By day 14 post infection, PD-L1 expression on these IEL was similar to control levels in C57BL/6 mice, but remained elevated by a mean of 1.5- and 1.9-fold in NOD and BALB/c mice, respectively (0.0012≤p≤0.0038). PD-L1 expression returned to control levels in all mice by day 21.

These data showed an association between the unaltered frequency of CD8αβ TCRαβ IEL in infant C57BL/6 mice and their low rates of gastrointestinal symptoms and infection. Similarly, the prolonged elevation of PD-L1 on the CD8αβ TCRαβ IEL of NOD and BALB/c mice related to the increased severity and duration of their diarrhoea, and elevated virus replication. In contrast, we found that RRV infection of 12 week-old NOD mice did not significantly alter the frequency, numbers and PD-L1 expression of CD8αβ TCRαβ IEL at days 2, 5 10 and 21 post infection (p>0.05; Fig. S1), consistent with the lack of diarrhoea and reduced intestinal RRV replication in these older mice [9].

### RRV titers in the MLN were highest in NOD mice

Infectious RRV is present in the pancreas, liver, spleen and blood of RRV-infected infant NOD mice, but other extraintestinal sites have not been analysed [8]. We examined the MLN for the presence of infectious RRV. Although a minority of MLN from all mouse strains contained infectious RRV at day 1 after infection (Fig. 2A), titres could not be determined due to low levels (Table 1). In NOD mice, most or all MLN on days 2 to 5 post infection contained RRV, with titers maximal at days 4 and 5 (Fig. 2A, Table 1). RRV spread to MLN was delayed in BALB/c mice, peaking at day 6 post infection when 6/8 MLN contained infectious RRV. This is consistent with previous studies where virus was detected by PCR [33]. Similarly, RRV titers were maximal on days 4 to 6 (Table 1). Infectious RRV was detected in 1/7 to 2/8 C57BL/6 mice on days 1 to 6 post infection, except for day 4 when the infection rate and virus titers peaked at 4/7 mice. MLN RRV titers in C57BL/6 mice remained low throughout the study (Table 1). C57BL/6 mice showed the least proportion of MLN with detectable virus titers, providing evidence that virus replicates to lower titers in these mice. RRV titers in the MLN were significantly greater in NOD than BALB/c and C57BL/6 mice on days 4 to 5 after infection (p = 0.035 and p = 0.040, respectively; Table 1).

### RRV infection increased T cell numbers in the MLN at day 2 after infection in NOD mice

The effect on RRV infection on MLN T cell numbers was determined. CD4^+^ T cell (Fig. 2B) and CD8αβ TCRαβ T cell (Fig. 2C) numbers increased over controls in the MLN of NOD mice at day 2 post infection (p = 0.0017 and p = 0.0025, respectively). However, in BALB/c and C57BL/6 mice these MLN cell numbers were similar between mock- and RRV-infected mice (p>0.05; Fig 2B, 2C). As the peak of RRV replication in MLN occurred at day 4 to 5 post infection, T cell numbers also were determined in RRV-infected mice at day 6 post infection. T cell numbers were similar in NOD and BALB/c mice, but were lower in C57BL/6 mice (Fig 2D). CD4^+^ rather than CD8^+^ T cell numbers were lower in RRV-infected C57BL/6 mice compared with the other mouse strains. The respective frequencies of CD8^+^ T cells expressing IFNγ at day 6 post infection (means±SD in NOD, BALB/c and C56BL/6 mice of 6.1±4.8%, 5.8±2.3% and 9.3±5.5%, respectively) did not differ (p>0.05; data not shown). Additionally, RRV-specific cells detected by IFNγ ELISPOT were rare in all three strains of mice, with 1 in 10^4^ mononuclear cells from MLN of RRV-infected mice stimulated with RRV VP7 5–13 peptide [32] secreting IFNγ (data not shown). This rate is similar to that seen previously with this peptide in BALB/c mice [34]. Overall, CD4^+^ and CD8^+^ T cell numbers were increased by RRV infection in NOD mice on days 2 and 6 post infection, BALB/c mice showed evidence of increased numbers on day 6, and T cell numbers were unaltered in C57BL/6 mice. No difference in the extent of CD8^+^ T cell activation between mouse strains was observed.

### Infectious RRV was present in the thymus of NOD, BALB/c and C57BL/6 mice during the first 5 days after infection

**Figure 3:**
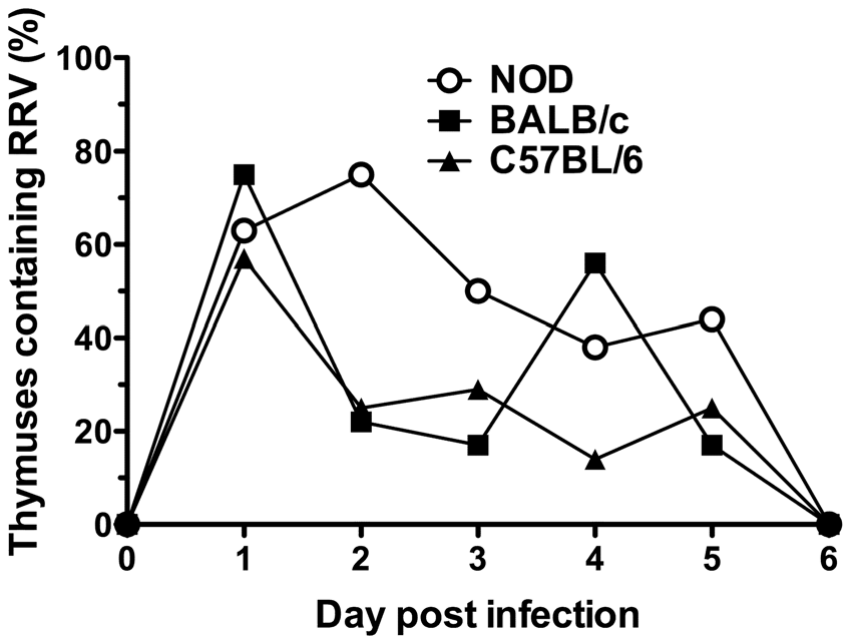
Thymic infection by RRV. On days 1, 2, 3, 4, 5 and 6 post infection, thymuses were assayed for the presence of infectious RRV by co-culture from 8, 8, 8, 8, 9 and 5 NOD mice, 8, 9, 6, 9, 6 and 8 BALB/c mice and 7, 8, 7, 7, 6 and 9 C57BL/6 mice, respectively.

RRV was found above to alter T cell numbers in the intestine and MLN, and can spread to other organs [8]. As the thymus is the source of the T cell repertoire, it was examined for the presence of RRV. The proportion of RRV-infected NOD mouse thymuses peaked at day 2 post infection (6/8) with titers ranging from 10 to 1500 FCFU/thymus (Fig. 3, Table 2). Biphasic intestinal rotavirus infection can occur in infant BALB/c mice [35], although this was not evident in our study (Fig. 1A, 2A). However, BALB/c mice showed biphasic thymus infection, with 6/8 thymuses containing infectious virus at 1 day post infection in the initial peak and a second peak when 5/9 thymuses were infected at day 4 (Fig. 3). RRV titers (FCFU/thymus) ranged from 380 to 11,000 on day 1 and 1,300 to 15,000 on day 4 post infection (Table 2). C57BL/6 infection was maximal day 1 post infection with 5/7 thymuses positive, containing titres of 17 to 3,200 FCFU. By day 2, RRV levels in the 2/8 positive thymuses were too low to be determined by titration (Table 2). Thymic RRV infection was restricted to 5 days for all mice, as it was undetectable on days 6, 10 and 14 post infection (Fig. 3 and data not shown).

**Table 2:** Titers of RRV in thymus homogenates from infant BALB/c, NOD and C57BL/6 mice.

Day post infection	Proportion of thymuses in the given mouse strain from which a virus titer was obtained, and the virus titer					
	NOD		BALB/c		C57BL/6	
	% of mice^1^	Virus titer^2^	% of mice	Virus titer	% of mice	Virus titer
1	25 (2/8)	2,100 (340–13,000)	75 (6/8)	2,400 (380–11,000)	71 (5/7)	170 (17–3,200)
2	75 (6/8)	390 (100–1,500)	22 (2/9)	500 (190–1,300)	0 (0/8)	NA^3^
3	38 (3/8)	410 (68–1,200)	17 (1/6)	190	29 (2/7)	880 (510–1,500)
4	25 (2/8)	110 (71–1,700)	56 (5/9)	3,500 (1,300–15,000)	14 (1/7)	1,500
5	0 (0/9)	NA	17 (1/6)	3,000	33 (2/6)	260 (170–410)

1 Percentage (no. giving a virus titer/no. collected) of mouse thymuses where an infectious virus titer could be determined. All organs where a titer was measurable also showed infectious virus by co-culture (Fig. 3).

2 Reciprocal geometric mean titer (range) of RRV in FCFU/thymus. No range is given when only a single thymus yielded a virus titer.

3 NA, not applicable. The infectious RRV level was below the limit of detection by titration.

### Nature and location of RRV-infected cells in the thymus

**Figure 4:**
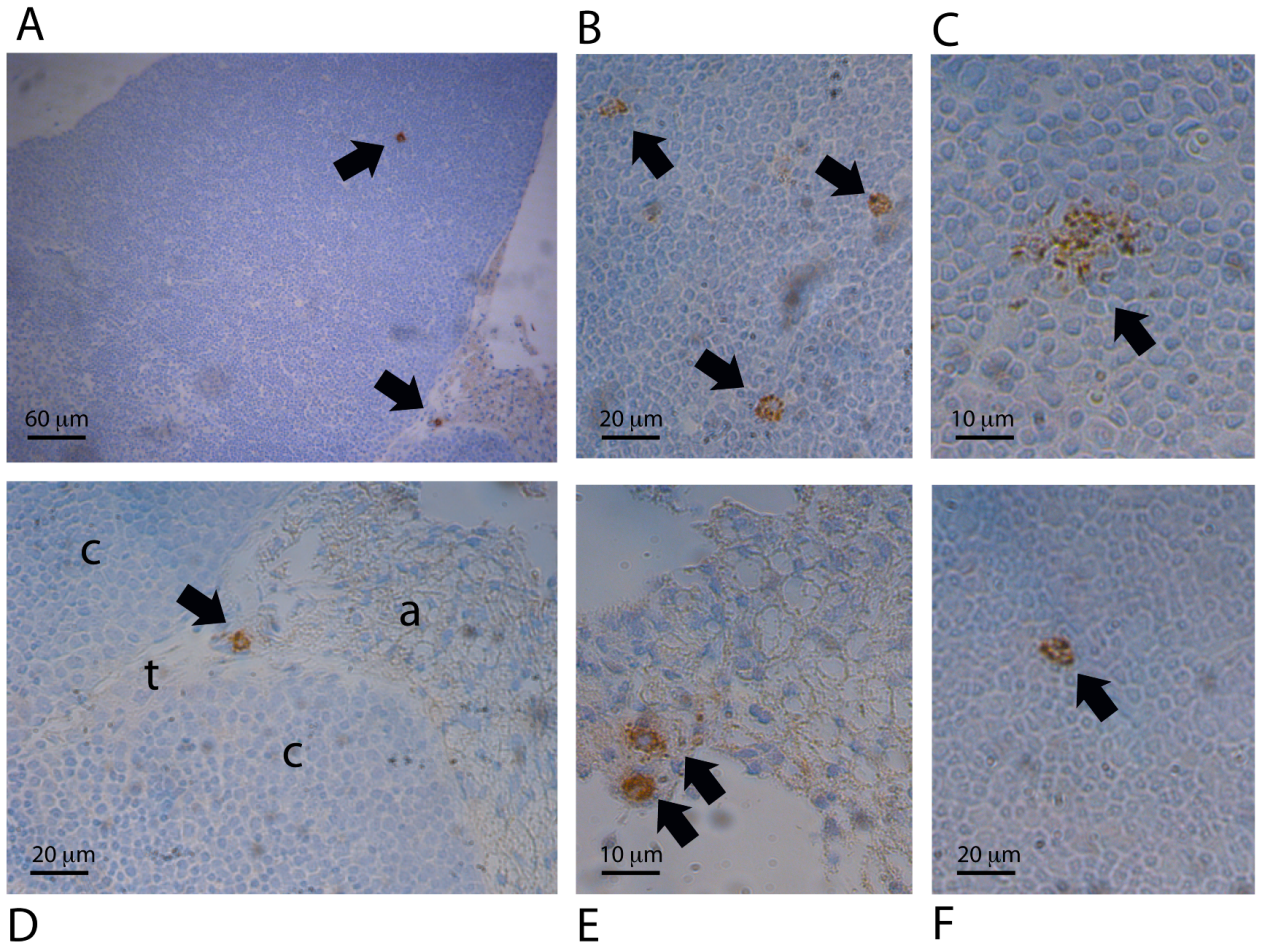
Identification of macrophages as the RRV-infected cells in the thymus, and their location. Thymus sections from 8 infants of each mouse strain were examined at the peak of thymic infection by immunohistochemistry. A, Representative section showing infected macrophages in the cortex and adipose tissue (×100). B, Three infected macrophages in the cortex (×300). (C) Infected macrophage in the cortex (×600). (D) Higher magnification (×300) image of an infected macrophage from A in the adipose tissue (a) near the trabeculae (t) and cortex region (c). (E) Two infected macrophages in the adipose tissue under the thymus capsule (×600). F, Rare infected macrophage in the cortex region of the NOD thymus (×300). All images except F are from thymuses of BALB/c mice at 4 days post infection. No cells positive for RRV were seen in matching sections stained with the negative control antibody, or in thymus sections from mock-infected mice of the same strain at the same day post-infection.

In sections of thymus obtained at the peak time of infectious RRV detection from NOD (day 2 post infection), BALB/c (days 1 and 4) and C57BL/6 (day 1) mice, RRV-infected cells were detected in the cortex and the adipose tissue under the capsule. Their morphology indicated that they were macrophages (Fig. 4). Numbers of infected macrophages were highest in BALB/c mice, with 1 to 4 seen per section in the cortex and adipose tissue on both days (Fig. 4A to 4E). Infected macrophages were less often observed in the cortex of NOD and C57BL/6 mice, and adipose tissue of C57BL/6 mice, and were rare in NOD adipose tissue (Fig. 4F). As positive and negative selection occur in the cortex, RRV-infected macrophages potentially could affect both these processes.

### RRV infection alters the thymic T cell repertoire

**Figure 5:**
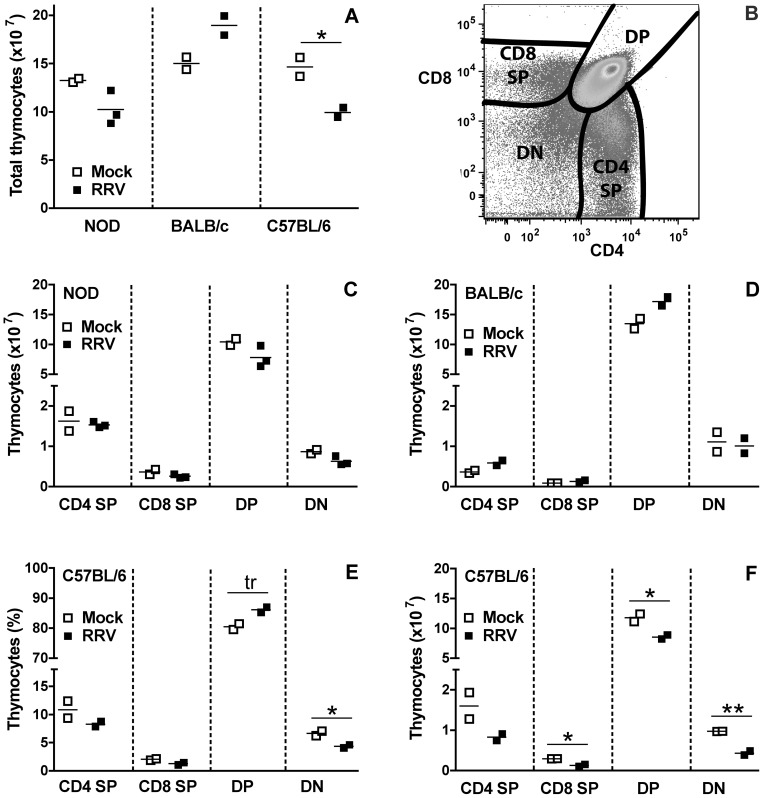
Effect of RRV on T cell populations in the thymus of mice at 14 days post infection. A, Total number of thymocytes obtained. B, Flow cytometry gating strategy for identification of the frequency of the CD4 SP, CD8 SP, DP and DN thymyocyte subsets. The absolute number of each thymyocyte subset in NOD and BALB/c analysed in A is shown in C and D, respectively. The frequency (E) and absolute number (F) of thymyocyte subsets in C57BL/6 mice analysed in A was determined by flow cytometry gating and cell counts, respectively. For A to F, each data point indicates results from a pool of cells from 2 to 4 mice, normalized to represent cells obtained/mouse. Cell pooling was necessary to obtain sufficient cells for analysis. Bar = mean. * 0.02 ≤ p ≤ 0.04; ** p = 0.008; tr  =  trend, p = 0.05.

The effect of RRV infection on thymocyte populations was determined after virus clearance. At day 7 post infection for all three mouse strains, no significant alterations in thymocyte numbers between mock- and RRV-infected mice were detected (p>0.05; data not shown). At day 14 (Fig. 5A) the total number of thymocytes isolated from RRV-infected NOD and BALB/c mice was unaltered compared to age-matched, mock-infected controls (p>0.05; Fig. 5A). In contrast, thymocyte numbers in C57BL/6 mice were significantly decreased over controls (p = 0.04; Fig. 5A).

During thymic T cell development, immature cells expressing neither CD4 nor CD8 (double negative; DN) evolve to express both CD4 and CD8 (double positive; DP) and undergo both positive and negative selection. The small minority of cells expressing a T cell receptor that binds MHC or peptide with at least a weak affinity are positively selected. DP cells with a high affinity for self-peptide or MHC undergo negative selection through apoptosis. DP cells with intermediate affinity can be converted into Treg (CD4^+^CD25^+^Foxp3^+^), and those with low affinity for self-antigen become Teff. At the end of the final, single positive (SP) stage of maturation, cells expressing either CD4 or CD8 located in the medulla are ready for release [36].

These stages of thymyocyte development were analysed by enumeration of CD4 SP, CD8 SP, DN and DP cells at day 14 post infection (Fig. 5B). The numbers and frequency of cells at each stage were not significantly altered by RRV infection of infant NOD and BALB/c mice (p>0.05; Fig. 5C, 5D and data not shown), consistent with their unchanged total thymyocyte numbers (Fig. 5A). In C57BL/6 mice (Fig. 5E), the proportion of DN cells was significantly decreased by RRV infection (p = 0.04), a trend for an increased proportion of DP cells was evident (p = 0.05) and CD8 SP cell proportions were unaltered (p>0.05). CD4 SP proportion and numbers were not reduced significantly by infection in these C57BL/6 mice (p = 0.14; Fig. 5E). However, their CD8 SP, DP and DN cell numbers were significantly decreased (p≤0.04; Fig. 5F), reflecting the decrease in total thymocyte numbers due to RRV infection (Fig. 5A).

**Figure 6:**
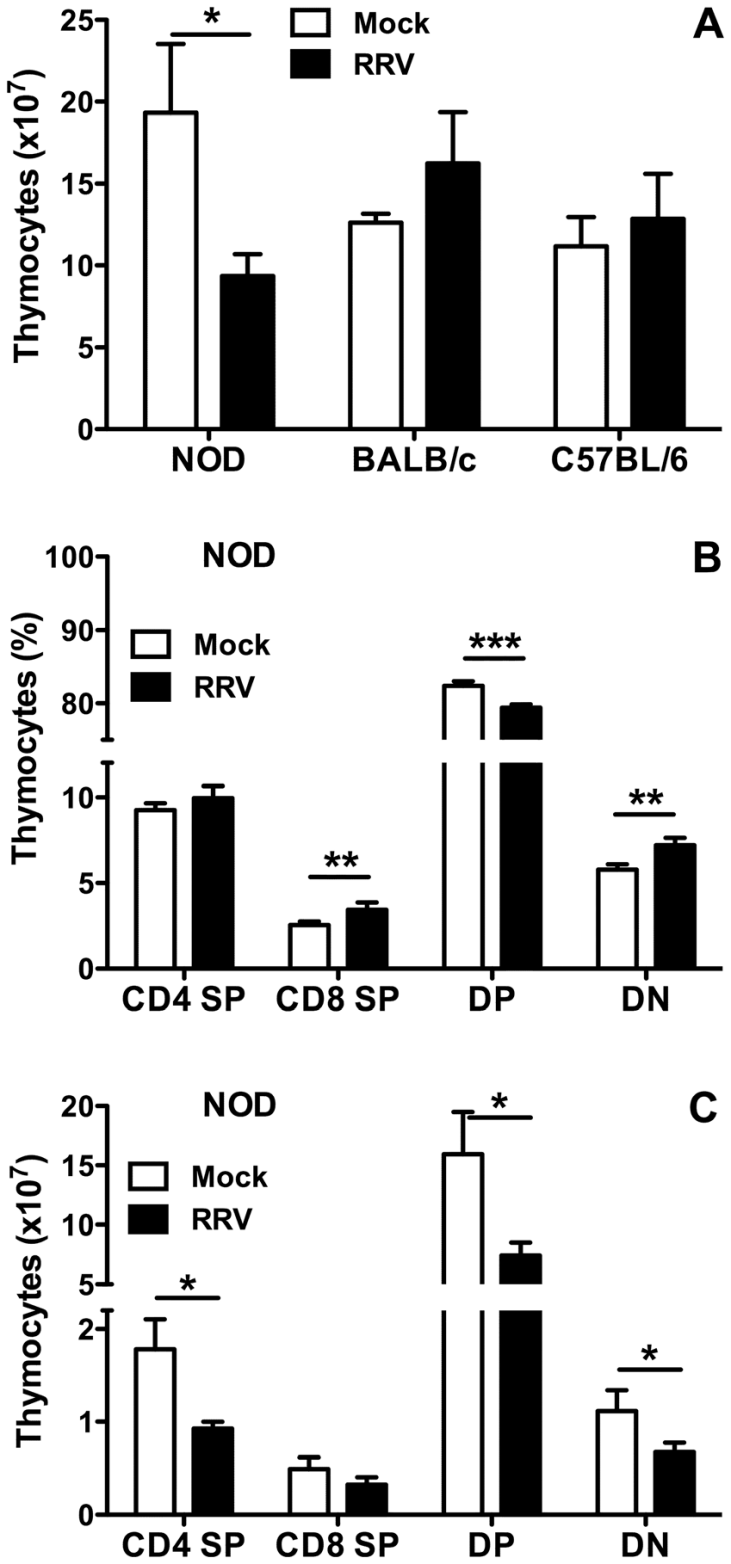
Effect of RRV on T cell populations in the thymus at 21 days post infection. A, Total number of thymocytes recovered from mock- and RRV-infected mice. For each inoculum, 4 (NOD), 3 (BALB/c) and 2 (C57BL/6) individual pools of cells were analysed. Each cell pool was produced from 2 (NOD), 2 to 3 (BALB/c) or 3 to 4 (C57BL/6) mice. Cell numbers from pools were normalized to represent cells obtained/mouse. The frequency (B) and absolute number (C) of thymocytes in the CD4 SP, CD8 SP, DP and DN thymocyte populations (defined in Fig. 5B) of the NOD mice described in A was determined by flow cytometry gating and cell counts, respectively. * p<0.05; ** p<0.01; *** p<0.001.

At day 21 post infection, total thymocyte numbers in NOD mice were significantly reduced over controls (p = 0.020; Fig. 6A). The DP cell frequency decreased, whereas CD8 SP and DN cell frequency increased (p≤0.0019), and the CD4 SP frequency was unaltered (p = 0.14, Fig. 6B). RRV infection significantly decreased the numbers of CD4 SP, DP and DN thymocytes in NOD mice at day 21 post infection (p≤0.020; Fig. 6C), and CD8 SP cells showed a possible trend for reduced numbers (p = 0.064; Fig. 6C). In contrast to NOD mice, total thymocyte numbers in BALB/c and C57BL/6 mice were similar to those of age-matched, mock-infected controls (p>0.11, Fig. 6A). As expected from these data, the thymocyte frequency and number for each developmental stage were similar between infected and control BALB/c or C56BL/6 mice at day 21 (data not shown).

### RRV infection decreased T cell numbers in the thymus of NOD mice, while Treg numbers were preserved

**Figure 7:**
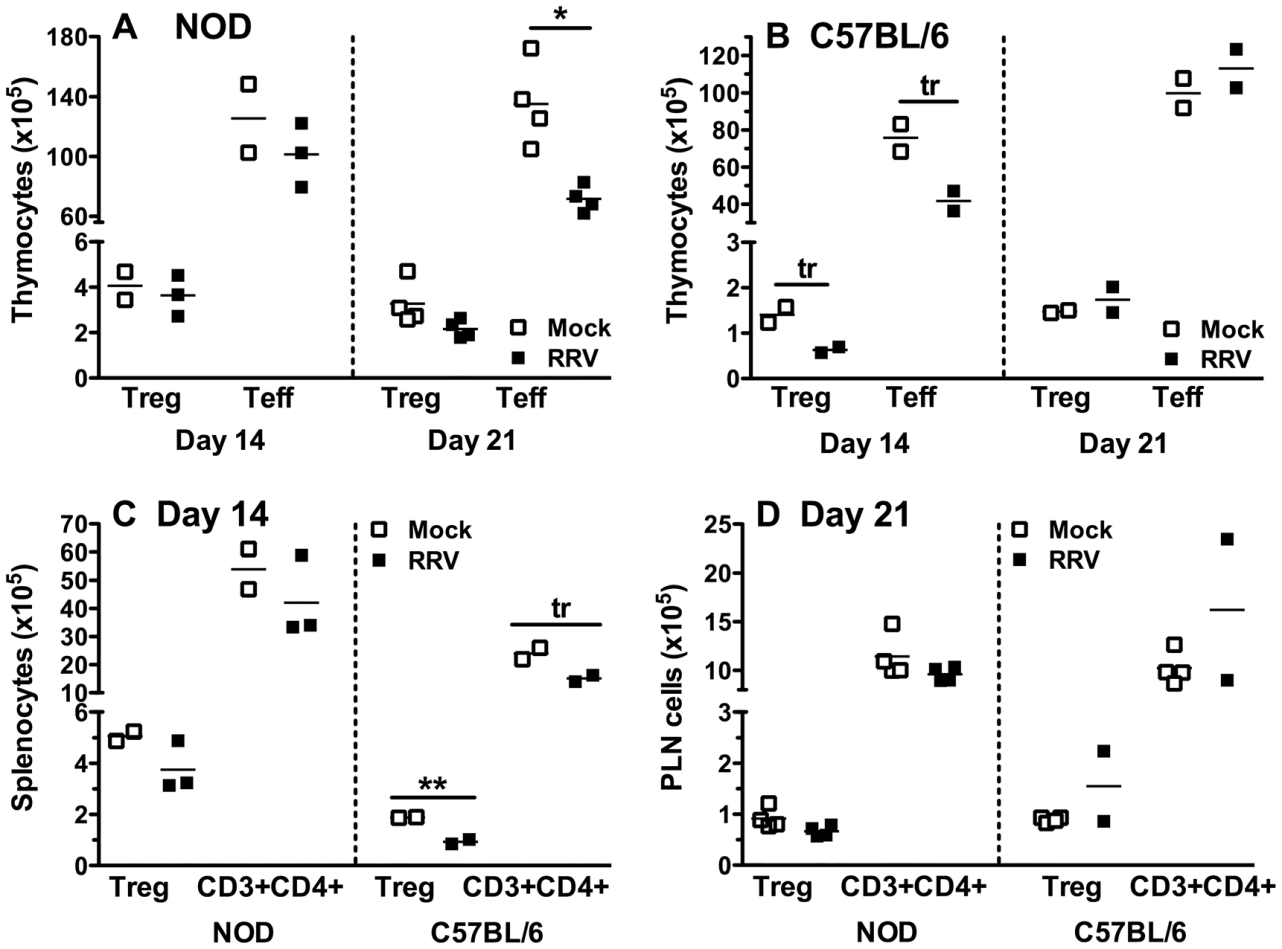
Thymic Treg numbers were maintained in NOD mice on days 14 and 21 after infection. Absolute numbers of thymic Treg (CD3^+^CD4^+^CD25^+^Foxp3^+^) and Teff (CD3^+^CD4^+^Foxp3^−^) cells in NOD (A) and C57BL/6 (B) mice on days 14 and 21 post infection. Each data point indicates results from a pool of cells from 2 to 4 mice, normalized to represent cells obtained from a single mouse. C, Decreases in total CD4^+^ T cell (CD3^+^CD4^+^) numbers, and in the Treg subset (CD3^+^CD4^+^CD25^+^Foxp3^+^) in the spleen (C) and PLN (D) of RRV-infected NOD and C57BL/6 mice compared with controls at days 14 (C) and 21 (D) post infection. Each data point represents a pool of cells from 4 to 5 mice, normalized to represent cells/mouse. Greater numbers of PLN cell pools than thymocyte and splenocyte pools were analysed from C57BL/6 mice, due to PLN availability from other studies. Bar = mean. * p = 0.02; ** p = 0.008; tr  =  trend, 0.05≤p≤0.06.

T cells (CD3^+^) in the CD4 SP population can be further divided into Treg that express CD25 and Foxp3, and Teff that lack Foxp3 expression. It was shown above that total thymocyte or CD4 SP cell numbers in infant NOD and C57BL/6 mice were reduced by RRV infection at days 14 or 21 after infection, the age when the thymus reaches its plateau in Treg production in infant mice [37]. Repeated transfer of Treg into infant NOD mice has been shown to delay their diabetes onset [38]. The effect of RRV infection on thymic production of Treg and Teff therefore was investigated. As expected from the unaltered CD4 SP cell number and frequency at day 14 after RRV infection in NOD mice (Fig. 5D), Treg and Teff numbers at this time also were unaffected by RRV infection (Fig. 7A). However, at day 21 post infection CD4^+^ Teff cell numbers were significantly reduced over controls in RRV-infected NOD mice (p = 0.022; Fig. 7A), whereas Treg numbers were unaltered (p = 0.079; Fig. 7A). Consistent with their unchanged thymyocyte levels on days 14 and 21 post infection (Fig. 5, 6), Treg and Teff numbers and frequency were unaltered in BALB/c mice at these times (p>0.05; data not shown). Treg and Teff numbers were unchanged at day 21 after RRV infection in C57BL/6 mice (Fig. 7B). In contrast, there was a trend for reduced Treg and Teff numbers at day 14 (p = 0.05 and p = 0.06, respectively; Fig. 7B). In order to determine if RRV infection induced similar effects on Treg and Teff cells in the periphery to those seen in the thymus, splenocytes at day 14 and PLN cells at day 21 after inoculation also were evaluated. Treg and CD3^+^CD4^+^ T cell numbers in RRV-infected NOD mouse spleens were similar to those in mock-infected mice (p>0.05; Fig. 7C), reflecting the thymus data. In C57BL/6 mice, similar reductions to those found in the thymus also were present in the spleen (p = 0.008 and p = 0.06, respectively; Fig. 7C). Neither NOD nor C57BL/6 mice showed altered Treg or CD3^+^CD4^+^ T cell numbers in the PLN at day 21 following RRV infection (p>0.05; Fig. 7D). Overall, preservation of thymic Treg during loss of thymic Teff CD4^+^ T cells at day 21 after NOD mouse infection resulted in an increased thymic Treg:Teff ratio. This alteration was not detected simultaneously in the PLN. This increased thymic Treg:Teff ratio may favor prolonged Treg suppression of self-reactive T cells once these Treg establish in the periphery.

### Adult NOD mice infected as infants produced PLN cells with reduced proliferative capacity in response to T cell stimulation

NOD mice have reduced Treg numbers [38] and the suppressive ability of their Treg wanes with age [39]. Reduced Teff suppression occurs in part through reduced Treg function and also by an increasing resistance of NOD Teff to suppression mediated by Treg [39], [40], [41]. NOD mice aged 10 weeks show extensive pancreatic infiltration by T and B lymphocytes, NK cells, dendritic cells and macrophages, which precedes diabetes onset from approx. 12 weeks of age [42]. The PLN is the priming site for T cells that migrate into the pancreas [43], [44].

**Figure 8:**
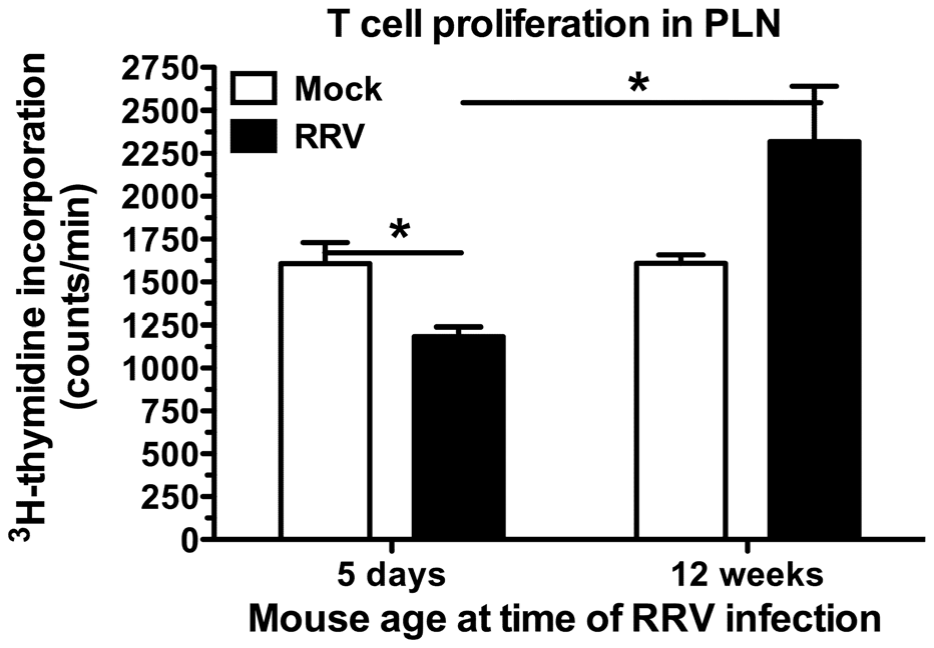
Reduced T cell proliferation by PLN cells from adult NOD mice infected as infants. PLN cells from groups of age-matched mice, which had been mock- or RRV-infected either as infants (5 days of age) or adults (12 weeks of age), were harvested at 17 weeks of age. Equivalent numbers of cells from mice in each group were stimulated with antibodies to CD3 and CD28. T cell proliferation was measured by ^3^H-thymidine incorporation (counts/min). Data for 4×10^5^ cells/well are shown. A pool of cells from 4 (infected as infant) or 5 (infected as adult) mice was analysed in triplicate for each inoculum. * p<0.05.

In order to determine the effect of RRV infection in infant mice on their T cell function as adults, the proliferative response by PLN cells was examined. Age-matched female NOD mice were mock- or RRV-infected as infants (5 days old) or adults (aged 12 weeks) and their PLN cell responses compared when they reached either 17 or 20 weeks of age. Regular testing showed that these mice were non-diabetic. The entire PLN cell population was used to best represent the lymphocytes present. T cell numbers in this population were similar between mock and RRV-infected mice, so results were not confounded by variation in input T cell number (data not shown). PLN cells from NOD mice that were RRV-infected as an infant showed significantly less proliferation in response to strong T cell stimulus at 17 weeks of age than mock-infected mice (p = 0.035; Fig. 8). In contrast, PLN cells from mice infected with RRV as adults were significantly more proliferative than those from mice infected as infants (P = 0.025; Fig. 8), in line with the accelerated diabetes onset observed in RRV-infected adult mice [9]. By 20 weeks of age, PLN T cell proliferation in mice infected as infants was similar between RRV-infected and control mice (data not shown), consistent with the previous demonstration that RRV infection as an infant delays but does not completely prevent diabetes [8]. It is possible that the waning in ability of Treg to suppress Teff that occurs in naïve NOD mice is delayed but not abolished by RRV infection. Overall, infection of infant NOD mice with RRV transiently decreased the proliferative ability of T cells in response to a strong T cell stimulus, most likely as the result of increased Treg function or decreased Teff resistance to Treg suppression.

## Discussion

Following RRV rotavirus infection as an infant, it is shown here that virus replication in the intestine and MLN is greater in diabetes-prone NOD mice than diabetes-resistant mouse strains. Furthermore, the extent of diarrhoea varies between mouse strains and is related to the degree of RRV replication in the intestine, MLN and thymus. Higher gastroenteritis rates also correlate with increased numbers of CD8^+^ IEL showing prolonged elevations in PD-L1 expression. We have demonstrated for the first time that RRV spreads to the mouse thymus and can alter T cell development. Although thymocyte production is reduced at 3 weeks after RRV infection in NOD mice, thymic Treg numbers are maintained. Furthermore, at 17 weeks of age in NOD mice given RRV as infants, PLN T cells show decreased proliferative ability in response to a strong stimulus. It is proposed that the maintenance of thymic Treg numbers at the time of reduced T cell production leads to this decreased T cell proliferative response in the PLN, which could contribute to the delayed diabetes onset in NOD mice following RRV infection as an infant.

IEL also accumulate during murine viral infection with reovirus [45], and intestinal T cell numbers are increased in rotavirus-infected calves [46]. RRV infection induces a rapid expansion of CD8αβ TCRαβ IEL in infant NOD mice (Fig. 1A, 1B) but not in adult NOD (Fig. S1) or C57BL/6 mice [47]. RRV infection had no effect on CD8^+^ TCRγδ IEL, which increased as a proportion of total T cells from 19±4% on day 14 post infection to 47±2% on day 21 and 59±1% on day 35 in both mock- and RRV-infected NOD mice (C. Zufferey and B. S. Coulson, unpublished data). These results confirm previous findings that the TCRγδ IEL frequency in mice reaches ∼50% by 20 days of age, and are consistent with the lack of effect of reovirus infection on TCRγδ IEL frequency compared to control mice [48], [49]. The low level of RRV replication in infant C57BL/6 mice may explain their unaltered numbers of CD8αβ TCRαβ IEL and rapid decrease in PD-L1 expression intestinally. The extensive RRV replication by day 1 post infection in NOD and BALB/c mice results in higher numbers of CD8αβ TCRαβ IEL to fight infection. Endothelial MHC class I levels are higher in NOD than BALB/c mice, influencing T cell proliferation, adhesion and transmigration of CD8^+^ T cells in NOD mice [50]. The elevated CD8αβ TCRαβ T cell numbers in the IEL compartment of NOD over BALB/c mice (Fig. 1C, 1D) could result from increased RRV antigen presentation on MHC class I.

The increased frequency of CD8αβ TCRαβ IEL and their extended PD-L1 expression is evident in BALB/c and NOD but not C57BL/6 mice, and might relate to the degree of virus replication. The increased PD-L1 expression at 2 days post infection suggests a role for PD-L1 in an enhanced immune response for clearance of rotavirus infection. PD-L1 interaction with PD-1 plays an important role in preventing autoimmune disease by inducing T cell anergy and tolerance [17]. However, in pathogen-specific T cell responses, PD-L1 is involved in T cell activation, proliferation and cytokine production [18], [19], [51]. In line with this latter PD-L1 role, our results show that PD-L1 expression correlates with the extent of intestinal rotavirus replication in all mouse strains. PD-L1 expression on CD8^+^ T cells in other tissues may prove to be a useful marker of rotavirus infection.

Our detection of RRV in thymic macrophages extends the earlier identification of macrophages as the likely main RRV source in the infant NOD mouse pancreas, and supports the proposal that macrophages disseminate rotavirus infection [8]. Altered thymic T cell development following infection by any virus that delays diabetes has not been reported to date. However, in an adult SJL/J mouse model of virus-induced diabetes, coxsackievirus B4 infection of the thymus, pancreas and other organs was associated with an increased DN thymocyte frequency but unaltered overall thymocyte numbers at 21 days post infection [52]. In fetal thymic organ cultures from CD-1 mice, coxsackievirus B4 increased the frequency of DN, CD4 SP and CD8 SP cells and decreased DP cells [53]. Thymic RRV infection in NOD mice also was associated with increased proportions of CD8 SP and DN, and decreased DP cells, although CD4 SP cell proportions were unaltered. However, total thymocyte numbers were decreased due to reductions in CD4 SP, DP and DN cells, in contrast to the coxsackievirus B4 studies [52], [53]. Thus, reduced overall thymyocyte numbers, combined with maintenance of CD4 SP cells, might contribute to the RRV-mediated diabetes delay. However, the effect of RRV on thymocytes is mouse strain-dependent, with most subsets and total cell numbers decreased in C57BL/6 mice, but no effect in BALB/c mice. The differing thymic effects of coxsackievirus and rotavirus in diabetes mouse models may relate to their contrasting effects on diabetes and the mouse strain utilized.

NOD mice show a normal ability to generate and maintain Treg in the thymus [54]. However, diabetes develops as Treg become functionally defective in the periphery with age, and self-reactive Teff increasingly develop resistance to Treg-mediated suppression [39], [40], [41]. We found that RRV-infected NOD mice maintain thymic Treg production at day 21 post infection when CD4^+^ Teff and other thymocyte subsets are reduced. At this time, the number and proliferative function of T cells in the PLN and MLN of mock- and RRV-infected NOD mice are similar (Fig. 7D and data not shown), as expected once infection is cleared and homeostasis has been restored. PLN T cells from NOD mice aged 17 weeks, which had been RRV-infected as infants, are less proliferative to a strong T cell stimulus than cells from age-matched, mock-infected mice. It is proposed that RRV infection of infant NOD mice may prolong Treg immunosuppression, which could contribute to their delayed diabetes onset. A lowering of Teff resistance to Treg-mediated regulation [40], [41] during RRV infection also may be a factor in this reduced T cell proliferation that occurs following a strong stimulus.

The wide range of immunological defects in NOD mice includes resistance to thymic deletion and increased production of autoimmune T cells [6], [7]. For example, DP thymocytes from NOD mice have a lower activation threshold than those of C57BL/6 and BALB/c mice [36]. Although we have associated Treg maintenance and decreased T cell proliferation after a strong T cell stimulus with the delayed diabetes after RRV infection, it is also possible that rotavirus infection of the NOD thymus induces stronger activation of the normally weakly activated self-T cells, leading to their deletion and contributing to the delayed diabetes onset. Further studies now are warranted to address the effects of rotavirus infection on thymic T cell activation, and the role of immunosuppression by Treg in the delayed diabetes mediated by RRV.

## Supporting Information

Figure S1 **Intestinal IEL responses following RRV infection of 12 week-old female NOD mice.** A, Frequency and B, absolute numbers of CD8αβ TCRαβ IEL in the intestine at days 2 to 21 after mock- or RRV infection. C, PD-L1 expression by CD8αβ TCRαβ IEL in the intestines of RRV-infected mice at days 2, 5 and 21 post infection. Data are expressed as the ratio of PD-L1 expression on cells from RRV-infected mice to the mean PD-L1 expression of mock-infected mice. Each data point represents a single mouse. Bar = mean.
(TIFF)
